# Clinical evaluation of AI Chatbots’ performance and safety for total parenteral nutrition planning in critically ill patients: a simulation-based benchmarking study

**DOI:** 10.3389/fnut.2026.1969789

**Published:** 2026-10-06

**Authors:** Sufyan Alomair

**Affiliations:** Department of Pharmacy Practice, College of Clinical Pharmacy, King Faisal University, Al-Ahsaa, Saudi Arabia

**Keywords:** artificial intelligence, ChatGPT, clinical decision support, Copilot, Gemini, parenteral nutrition, patient safety, Perplexity

## Abstract

**Introduction:**

Total parenteral nutrition (TPN) is essential for critically ill adults unable to meet their nutritional needs via the gastrointestinal tract. Recent advancements in large language models (LLMs) have sparked interest in their potential to support healthcare providers in complex clinical decisions. Nonetheless, no prior research has systematically compared publicly accessible chatbots to pharmacist-developed reference TPN plans across a diverse patient cohort, using a predefined, guideline-based rubric assessing accuracy, completeness, safety, error types, and numerical precision. This study presents a simulation-based benchmarking to evaluate (i) the accuracy and comprehensiveness of chatbot-generated TPN plans, (ii) their safety, acceptability, and error profiles, and (iii) the numerical agreement and concordance with expert pharmacist references.

**Methods:**

This benchmarking study, based on simulations and cross-sectional analysis, evaluated the performance of four publicly accessible AI chatbots—ChatGPT, Gemini, Copilot, and Perplexity—in formulating TPN plans for critically ill adults. Copilot declined to generate patient-specific TPN plans; this refusal was retained as a performance outcome, while Copilot was excluded from analyses requiring an analyzable TPN prescription. Each chatbot produced one plan for each of 10 standardized clinical cases, resulting in a total of 30 TPN plans. Friedman tests, Kendall’s *W*, and Holm-adjusted Wilcoxon signed-rank tests were used for comparative analyses.

**Results:**

ChatGPT produced comprehensive plans for all 10 cases, compared to 6 plans from Gemini and 4 from Perplexity. The median accuracy was 57.7% for ChatGPT, 43.9% for Gemini, and 38.5% for Perplexity, with no statistically significant overall difference (*p* = 0.124). However, ChatGPT performed better than Perplexity in pairwise comparison (adjusted *p* = 0.035). There was a significant difference in completeness among the chatbots (*p* = 0.003; Kendall’s *W* = 0.57), with median scores of 64.6, 41.9, and 32.3%, respectively. All 30 plans had at least one major or critical safety issue, 7 (23.3%) contained a critical concern, and 19 (63.3%) were rejected. The most common errors were related to electrolytes (90.0%), micronutrients (70.0%), and monitoring (66.7%). Recommendations from chatbots often exceeded pharmacist-derived values, with median relative differences of +47% for energy, +120% for lipids, and +100% for potassium. Bland–Altman analysis revealed considerable variability in the agreement.

**Conclusion:**

ChatGPT produced the most comprehensive TPN plans among chatbots. However, none showed enough accuracy, safety, or concordance for independent use. All plans had critical issues, especially with electrolytes, micronutrients, monitoring, and refeeding precautions. AI chatbots can support education or planning but require review by a well-trained clinician before clinical use.

## 1 Introduction

Total parenteral nutrition (TPN) is a life-sustaining therapy for critically ill adults who cannot meet their nutritional needs via the gastrointestinal tract; however, it remains one of the most complex and high-risk interventions in clinical nutrition. Creating an appropriate regimen involves carefully balancing energy and protein goals, glucose infusion rate, lipid dose, fluid status, and a comprehensive range of electrolytes, trace elements, and vitamins. The process must also consider organ dysfunction, refeeding syndrome, and changing metabolic needs (1). Moreover, inappropriate regimens may exacerbate catabolism, impair immune function, delay recovery, and worsen clinical outcomes (2). Accordingly, parenteral nutrition is considered a high-alert therapy, and safe prescribing generally requires multidisciplinary assessment and ongoing clinical review (3, 4).

Clinical pharmacists play a pivotal role in ensuring the safety and appropriateness of PN prescriptions by verifying nutrient composition, compatibility, stability, electrolyte dosing, and ongoing monitoring (5). Current ASPEN and ESPEN guidelines also highlight that nutrition support in critically ill patients should be individualized and reassessed according to the patient’s evolving clinical condition (6, 7).

This is especially crucial in complex cases such as renal replacement therapy, fluid restriction, electrolyte imbalances, and refeeding risk, where nutritional and electrolyte needs can vary significantly between patients (2).

Recent advances in large language models (LLMs) have sparked significant interest in their ability to support healthcare professionals with complex clinical decisions. Their conversational interface, rapid response generation, and broad accessibility have driven greater interest in using them as clinical decision-support tools (8, 9). In clinical nutrition, chatbots show reasonable performance for general dietary advice and patient education, yet can produce confident, fluent, and incorrect or unsafe recommendations that vary with model and phrasing (10, 11). Prescribing a well-designed TPN regimen is far more demanding than answering a dietary question: it requires numerically specific, internally consistent, clinical judgments, and safety-critical output.

Although previous studies have evaluated LLM performance in medical exams, guideline interpretation, and medication tasks, evidence on their ability to produce comprehensive, clinically appropriate TPN prescriptions for critically ill adults. To our knowledge, no study has compared multiple publicly available chatbots against pharmacist-derived reference TPN plans across a range of critically ill patients using a predefined, guideline-based rubric that assesses accuracy, completeness, safety, error taxonomy, and numerical agreement. Consequently, we performed a simulation-based benchmarking study to (i) evaluate the accuracy and completeness of chatbot-generated TPN plans, (ii) examine their safety, acceptability, and error types, and (iii) assess their numerical concordance and agreement with a reference from an expert pharmacist.

## 2 Methods

### 2.1 Study design

This cross-sectional, simulation-based benchmarking study evaluated the ability of four publicly available AI chatbots—ChatGPT, Gemini, Copilot, and Perplexity—to create total parenteral nutrition (TPN) plans for critically ill adults (see Table 1). Copilot declined to generate a patient-specific TPN plan using the standardized prompt. This refusal was retained as a performance outcome; however, because no analyzable TPN prescription was generated, Copilot was excluded from subsequent plan-level and quantitative analyses. Each chatbot produced one plan for each of 10 standardized clinical cases, for a total of 30 AI-generated TPN plans. The study used only simulated cases, with no actual patients or identifiable data involved. The study assessed rubrics based on accuracy, completeness, clinical safety, acceptability, numerical reporting, and quantitative concordance with pharmacist reference plans. The predefined evaluation rubric is available in Supplementary Table S6. The classifications for clinical error severity ranged from none, minor, moderate, major, to critical. ‘No clinically significant error’ indicated the recommendation aligned with evidence-based TPN practices. Minor errors were unlikely to impact patient safety, whereas moderate errors needed pharmacist correction but were not expected to cause serious harm. Major errors could lead to significant harm if left uncorrected, and critical errors had the potential to cause life-threatening complications, permanent injury, or death.

**Table 1:** AI chatbots characteristics.

Chatbot Name	Developer	Model	Version used	Accessed date
ChatGPT	OpenAI	GPT-5.3	Free	27 June 2026
Copilot	Microsoft	NA	Free	27 June 2026
Gemini	Google	3.5 Flash	Free	27 June 2026
Perplexity	Perplexity	NA	Free	27 June 2026

### 2.2 Clinical case development and reference plans

Ten standardized adult ICU cases developed by the study investigator requiring TPN (with 30 TPN plans generated) covered a wide range of clinically relevant scenarios, including renal replacement therapy, fluid restriction, electrolyte imbalances, gastrointestinal dysfunction, high-output losses, refeeding risk, obesity, and hepatic issues. Each case included demographics, anthropometrics, medical history, lab results, fluid balance, and other data needed for the prescription. Claude (Anthropic) was used as a supportive tool during case development to help ensure inclusion of a broad range of relevant clinical characteristics; however, all cases were reviewed and finalized by the investigator. The cases were not intentionally designed as difficult or adversarial edge cases. These cases were developed independently of any chatbot included in the study to avoid circular bias and ensure unbiased evaluation. The complete clinical cases are provided in Supplementary Table S5.

Additionally, all reference TPN plans were independently developed by the study investigator, an experienced critical care pharmacist with expertise in parenteral nutrition. The reference plans were developed in accordance with the most recent ASPEN and ESPEN guideline recommendations and finalized before evaluating any chatbot-generated TPN plans to minimize potential bias. The same investigator subsequently evaluated all chatbot-generated plans using a predefined data-collection instrument assessing accuracy, completeness, clinical safety, acceptability, and error types, in accordance with the most recent guideline recommendations. These plans specified daily energy intake (initial and target), protein, dextrose, glucose infusion rate (GIR), lipids, total fluid volume, electrolyte levels, trace elements, multivitamins, infusion schedules, and monitoring protocols. The pharmacist-derived reference TPN plans and the corresponding AI-generated TPN recommendations for all 10 standardized cases are provided in Supplementary Table S1.

### 2.3 AI Chatbot evaluation

Three AI chatbots—ChatGPT (OpenAI), Gemini (Google), and Perplexity (Perplexity AI)—were assessed via their free web interfaces on 27 June 2026. The model’s name and version, access date, and subscription status were documented at the time of data collection. Each chatbot received the same clinical data and a standardized prompt: “Formulate a total parenteral nutrition plan for this patient according to ASPEN/ESPEN guidelines,” and each prompt was provided in English using the “new chat” feature. No follow-up questions or prompt changes were allowed after the initial responses, which were recorded exactly as produced, resulting in 30 plans (10 plans for each chatbot) that were subsequently evaluated according to the most recent ASPEN and ESPEN guideline recommendations, using the predefined data-collection instrument to improve consistency and reduce subjectivity. Parameters such as temperature and other sampling settings were not available through the publicly accessible web interfaces and therefore could not be standardized or reported.

### 2.4 Data extraction

Data were collected from each chatbot response via a standardized electronic form. This encompassed whether a comprehensive, patient-specific plan was generated; response length; total energy, protein, dextrose, glucose, lipid, and total fluid infusion rates; individual electrolyte doses; multivitamins; trace elements; acid–base considerations; infusion-related recommendations; monitoring parameters; and other pertinent clinical instructions. When a chatbot provided a numerical range instead of a single value, the midpoint was utilized for the quantitative analysis of concordance. Parameters that were not explicitly reported were deemed missing and excluded from parameter-specific concordance calculations. Their omission was separately addressed in the evaluations of completeness and numerical reporting.

### 2.5 Outcomes and scoring

#### 2.5.1 Accuracy

It was assessed using a binary rubric based on ASPEN/ESPEN guidelines, evaluating whether caloric targets, protein intake, fluid volume, dextrose/GIR, lipids, electrolytes (sodium, potassium, magnesium, calcium, phosphate), trace elements, multivitamins, and acid–base balance criteria were met (1 = criterion met, 0 = not met). The acceptable ranges used for accuracy assessments are available in Supplementary Table S7. For each AI-generated plan, the total accuracy percentage was calculated as:

$$\begin{array}{l} \text{Accuracy score}\;\;(\%)\\ =\frac{\text{Number of applicable accuracy criteria}\;\mathrm{met}}{\text{Total number of applicable accuracy criteria}}\times 100 \end{array}$$

Higher scores indicated greater adherence to the predefined clinical criteria.

#### 2.5.2 Completeness

It was measured by whether each essential component—such as macronutrients, electrolytes, micronutrients, fluid volume, refeeding precautions, insulin, fluid restrictions, infusion rate, and monitoring—was present (1) or absent (0). Items not applicable to a specific case were marked as such and excluded from that plan’s total; both accuracy and completeness were expressed as the percentage of applicable items fulfilled. The completeness percentage was calculated as:

$$\begin{array}{l} \text{Completeness score}\;\;(\%)\\ =\frac{\text{Number of applicable components reported}}{\text{Total number of applicable components}}\times 100 \end{array}$$

A separate binary variable recorded whether the chatbot generated a complete patient-specific TPN plan rather than partial recommendations.

#### 2.5.3 Clinical safety

Each plan was assessed for safety concerns that could have significant clinical consequences if implemented without pharmacist review. The evaluation was done per plan, categorizing concerns as no significant concern, minor, moderate, major, or critical, based on the highest severity concern identified. A major concern indicates an error or omission that could lead to clinically significant harm or require substantial correction before use. A critical concern is an error that has the potential to cause life-threatening complications or death if not corrected.

#### 2.5.4 Clinical acceptability

Plans were categorized as acceptable without modification, acceptable after minor modification, or rejected. A plan was rejected if it contained clinically significant deficiencies, unsafe recommendations, or omissions that required substantial pharmacist intervention before clinical use. In addition, a structured clinical-error taxonomy classified each plan’s deficiencies into domains (electrolytes, micronutrients, monitoring, refeeding, fluid, calories, dextrose, lipids). Recurrent high-consequence patterns were tabulated from the reviewer’s free-text error descriptions.

#### 2.5.5 Quantitative concordance with the pharmacist-derived reference plan

Quantitative concordance with the reference was computed for total energy, protein (g/day and g/kg/day), dextrose, lipids (g/day and g/kg/day), fluid volume, and each electrolyte, using absolute and relative differences. The absolute difference was calculated as:

Absolute difference=AIvalue−Pharmacist reference value

The relative difference was calculated as:

Relative difference(%)=AIvalue−Pharmacist reference valuePharmacist reference value×100

Positive values indicated that the chatbot’s recommendation was higher than the pharmacist reference, while negative values indicated it was lower. Relative differences were not calculated if the reference value was zero. When a range was reported by the chatbot, its midpoint was used; missing AI parameters were treated as missing. Agreement for energy, protein, and potassium was also evaluated, and concordance was interpreted descriptively as numerical agreement rather than evidence of clinical accuracy, appropriateness, or safety.

### 2.6 Statistical analysis

Continuous outcomes were presented as the median (interquartile range) and the mean with 95% CI, while categorical outcomes were shown as counts and proportions. The Shapiro–Wilk test was used to assess normality of quantitative variables. Accuracy, completeness, and response length were evaluated using the Friedman test and Kendall’s W. *Post hoc* pairwise comparisons were conducted using Wilcoxon signed-rank tests with Holm correction across the three pairwise comparisons. Safety severity and clinical acceptability outcomes were summarized descriptively. Bland–Altman analysis was used to assess agreement between chatbot-generated and pharmacist-derived values for energy, protein, and potassium. Given the limited number of observations, particularly for potassium, the Bland–Altman findings were interpreted cautiously. A two-sided *p* < 0.05 was considered significant. All analyses were performed in IBM SPSS Statistics, Version 32 (IBM Corp., Armonk, NY, USA).

## 3 Results

### 3.1 Plan generation and response characteristics

All chatbots responded to every case, excluding Copilot. Complete, patient-specific plans were generated in 10 out of 10 cases with ChatGPT, 6 out of 10 with Gemini, and 4 out of 10 with Perplexity. Response lengths varied significantly (Friedman *χ*^2^ = 9.8, *p* = 0.007; Kendall’s *W* = 0.49), with ChatGPT providing the most concise responses (median 112 words) and Gemini the longest (median 153 words). The difference between ChatGPT and Gemini was statistically significant (Holm-adjusted *p* = 0.006).

Copilot did not produce a patient-specific TPN plan for any of the 10 clinical cases when using the standard prompt. Its refusal was thus documented as a performance outcome. Since there were no TPN prescriptions to analyze, Copilot was excluded from later assessments of accuracy, completeness, safety, acceptability, error taxonomy, and numerical concordance.

### 3.2 Accuracy and completeness

ChatGPT achieved the highest scores, while Perplexity scored the lowest in both domains (Table 2 and Figure 1). The median accuracy was 57.7% (ranging from 47.9 to 66.7%) for ChatGPT, 43.9% (30.8 to 68.8%) for Gemini, and 38.5% (32.7 to 50.0%) for Perplexity. The overall differences were not statistically significant (*p* = 0.124; Kendall’s *W* = 0.21). However, *post hoc* analysis showed that ChatGPT outperformed Perplexity (Holm *p* = 0.035; rank-biserial r = 0.85).

**Table 2:** Rubric-based performance and response length by chatbot.

Metric	ChatGPT	Gemini	Perplexity	Friedman *P*	Kendall’s *W*
Accuracy, %—median (IQR)	57.7 (47.9–66.7)	43.9 (30.8–68.8)	38.5 (32.7–50.0)	0.124	0.21
Accuracy, %—mean (95% CI)	55.8 (47.0–64.7)	49.5 (35.3–63.7)	37.9 (28.2–47.7)		
Completeness, %—median (IQR)	64.6 (59.1–73.7)	41.9 (32.8–60.6)	32.3 (26.3–48.6)	0.003	0.57
Completeness, %—mean (95% CI)	66.7 (59.6–73.8)	48.0 (34.1–61.9)	36.1 (26.4–45.9)		
Response length, words—median (IQR)	112.0 (106.0–128.5)	153.0 (120.0–177.8)	122.5 (113.3–180.8)	0.007	0.49

IQR, interquartile range; CI, confidence interval.

**Figure 1:**
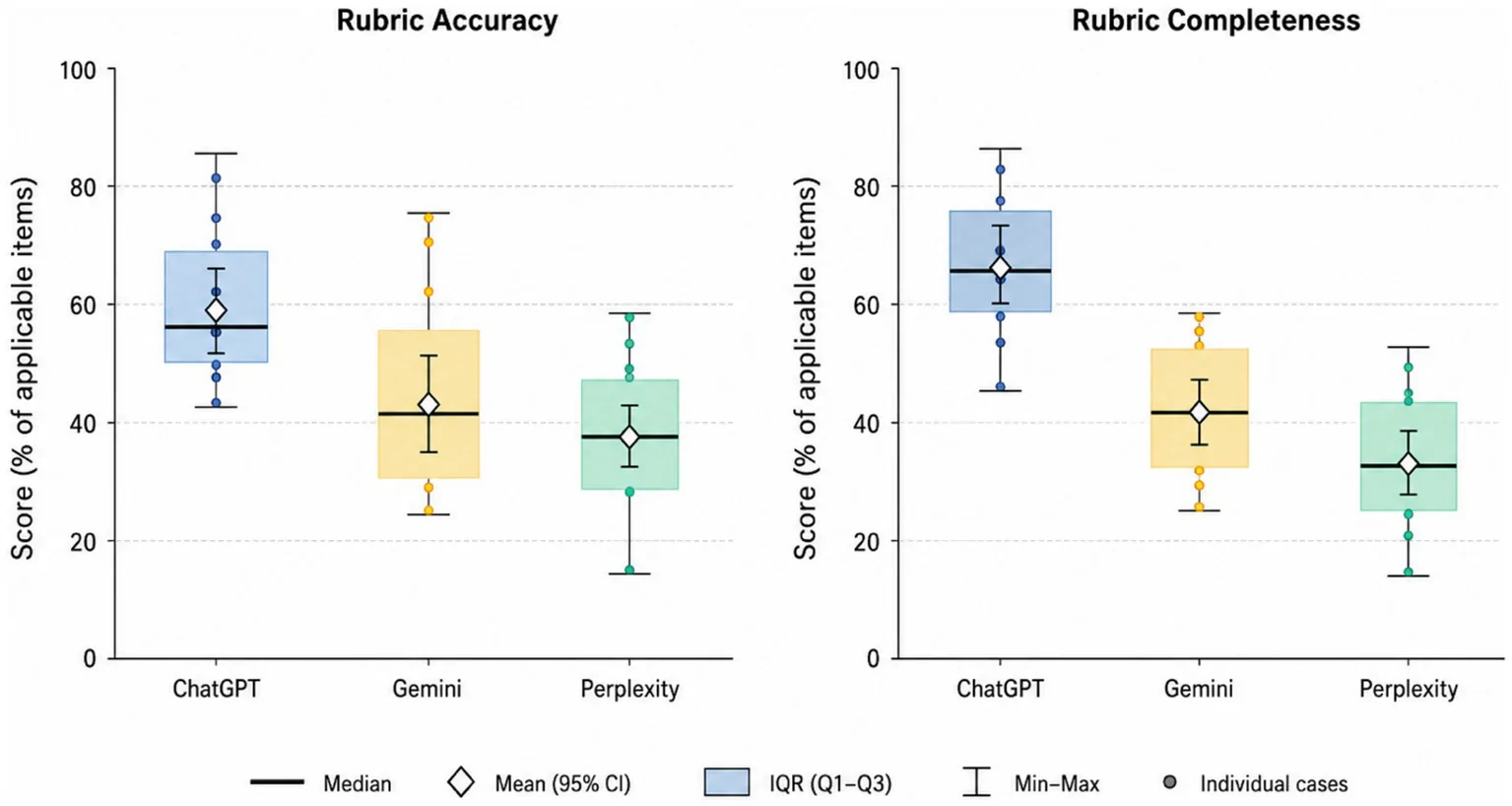
Rubric-based accuracy and completeness scores by chatbot.

Completeness showed a clearer difference (*p* = 0.003; Kendall’s *W* = 0.57, indicating a large effect size): the median completeness was 64.6% (range 59.1–73.7%) for ChatGPT, compared to 41.9% (32.8–60.6%) and 32.3% (26.3–48.6%) for the other methods. ChatGPT was significantly more complete than Perplexity (Holm *p* = 0.006; rank-biserial r = 1.00) (see Table 3).

**Table 3:** Pairwise comparisons between AI chatbots.

Comparison	Accuracy P (*r*)	Completeness P (*r*)	Response length P (*r*)
ChatGPT vs Gemini	0.563 (0.33)	0.160 (0.64)	0.006 (−1.00)
ChatGPT vs Perplexity	0.035 (0.85)	0.006 (1.00)	0.160 (−0.64)
Gemini vs Perplexity	0.523 (0.42)	0.160 (0.64)	0.510 (0.25)

Pairwise comparisons were performed using Holm-adjusted Wilcoxon signed-rank tests. *r*, rank-biserial correlation coefficient.

### 3.3 Domain-level accuracy

The TPN plans showed the strongest results in macronutrient targets, with protein, dextrose/GIR, and lipid doses deemed appropriate in most cases across all chatbots (Figure 2). Conversely, electrolyte and micronutrient prescribing emerged as the main areas of failure: the correct dosing of sodium, potassium, calcium, and phosphate was frequently unmet, and trace-element and multivitamin dosing was nearly always incorrect—appropriate in only 2 out of 30 plans for trace elements and 1 out of 30 for multivitamins.

**Figure 2:**
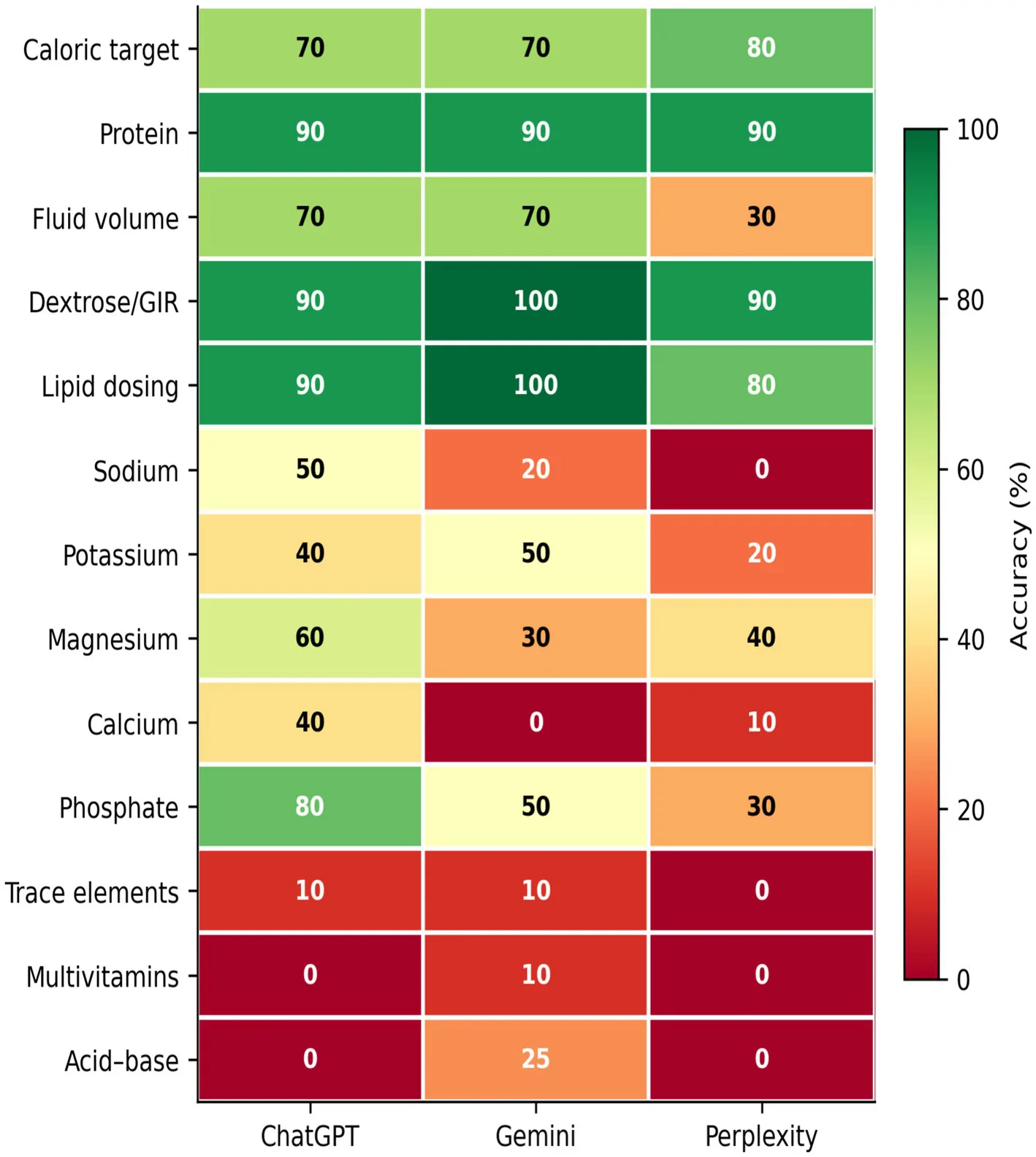
Domain-level accuracy across total parenteral nutrition parameters by chatbot.

### 3.4 Clinical safety and acceptability

Among the 30 evaluated TPN plans, the highest error severity was major in 23 plans (76.7%) and critical in 7 plans (23.3%) (Table 4). Thus, all 30 plans (100%) had a highest error severity classified as either major or critical. Critical errors, defined as those with the potential to cause life-threatening complications, permanent harm, or death if implemented without pharmacist review or clinical intervention, occurred in 7/30 plans (23.3%). Critical concerns were most frequent with ChatGPT (3/10) and equally frequent with Gemini and Perplexity (2/10 each). Only 3/30 plans (10%) were acceptable without modification; 19/30 (63%) were rejected; and 8/30 were acceptable after minor modification. ChatGPT had the fewest rejections (3/10), and Perplexity the most (9/10).

**Table 4:** Safety severity and clinical acceptability of AI-generated TPN plans by chatbot.

Outcome	ChatGPT	Gemini	Perplexity	Total
Fully complete plan generated	10	6	4	20
Highest severity = Major	7	8	8	23
Highest severity = Critical	3	2	2	7
Acceptable without modification	1	1	1	3
Acceptable after minor modification	6	2	0	8
Rejected	3	7	9	19

### 3.5 Clinical error taxonomy

The classification of clinical errors (Table 5 and Figure 3) revealed that most deficiencies were in electrolyte management (27 out of 30 plans, 90.0%), micronutrient supplementation (21 out of 30 plans, 70.0%), and monitoring recommendations (20 out of 30 plans, 66.7%). Errors related to refeeding precautions were found in 11 plans (36.7%), while errors involving fluid volume, dextrose, and lipid recommendations were less common. Similar error patterns were found across ChatGPT, Gemini, and Perplexity (see Table 6).

**Table 5:** Clinical error taxonomy.

Error domain	ChatGPT	Gemini	Perplexity	Total
Electrolytes	9	8	10	27
Micronutrients	7	7	7	21
Monitoring	5	8	7	20
Refeeding	4	4	3	11
Fluid	2	0	1	3
Dextrose	0	0	1	1
Lipids	0	0	1	1

Each plan may contribute to more than one domain.

**Figure 3:**
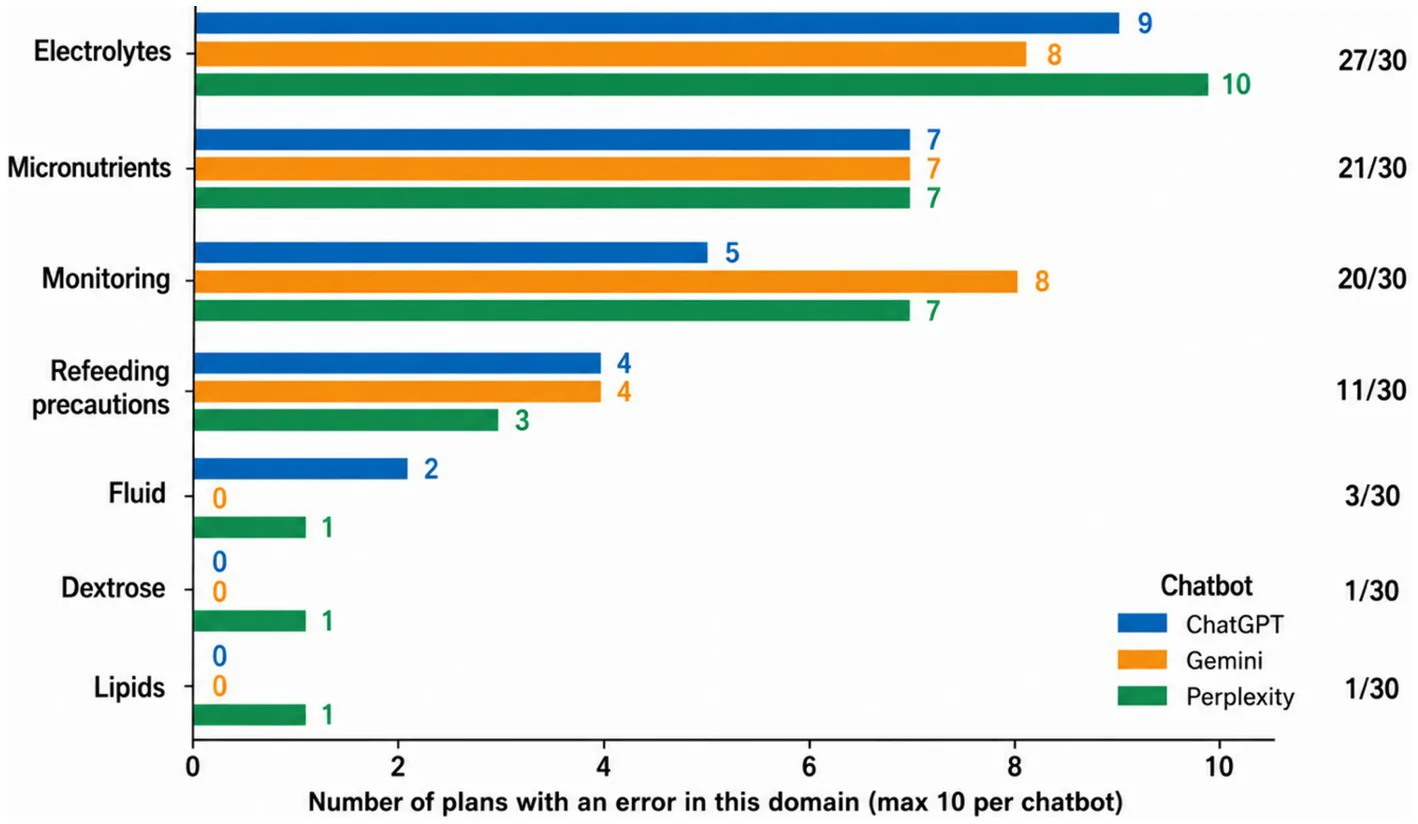
Clinical error taxonomy.

**Table 6:** Recurrent clinically significant error patterns identified in AI-generated TPN plans.

Pattern	ChatGPT	Gemini	Perplexity	Total
Missing trace elements/multivitamins	6	6	7	19
Missing monitoring plan	4	5	5	14
Missing refeeding-syndrome precautions	4	5	4	13
Missing/incomplete electrolyte doses	0	6	5	11
High-dose potassium in renal impairment/oliguria/dialysis	3	1	2	6
Excessive fluid volume	2	0	0	2

### 3.6 Quantitative concordance with the pharmacist-derived reference plans

Quantitative concordance between AI-generated recommendations and pharmacist-derived reference plans. The data show that, for most parameters, chatbot recommendations tend to exceed the pharmacist’s reference values, particularly for total energy, lipids, and potassium. The combined median relative differences were +47% for total energy, +120% for lipids, and +100% for potassium. Conversely, the smallest differences were observed for total fluid volume and dextrose, with median discrepancies generally within ±14% of the reference values (see Table 7).

**Table 7:** Median relative difference from the pharmacist reference (%).

Parameter	ChatGPT	Gemini	Perplexity	*n* reported	Within ±20% (%)
Total energy (kcal/day)	+50	+49	+30	30/30	20
Protein (g/day)	+38	+28	+26	30/30	10
Dextrose (g/day)	+31	+13	+14	29/30	41
Lipids (g/day)	+200	+100	+120	29/30	12
Total fluid volume (mL/day)	+10	+4	—	19/30	47
Sodium (mEq/day)	+41	+29	—	10/30	10
Potassium (mEq/day)	+180	+50	+88	20/30	5
Magnesium (mEq/day)	+71	0	+17	17/30	24
Calcium (mEq/day)	+25	−90	−13	7/30	43
Phosphate (mmol/day)	+67	0	+17	19/30	50

“+” over-prescription, “−” under-prescription, “—” not reported.

Bland–Altman analysis (Figure 4) revealed a consistent positive bias across several parameters. The average bias was +510 kcal/day for total energy (95% limits of agreement [LoA], −431 to +1,451 kcal/day), +24 g/day for protein (95% LoA, −62 to +111 g/day), and +40 mEq/day for potassium (95% LoA, −9 to +90 mEq/day). The wide limits of agreement suggest considerable variability among clinical cases, despite the bias remaining in the same direction.

**Figure 4:**
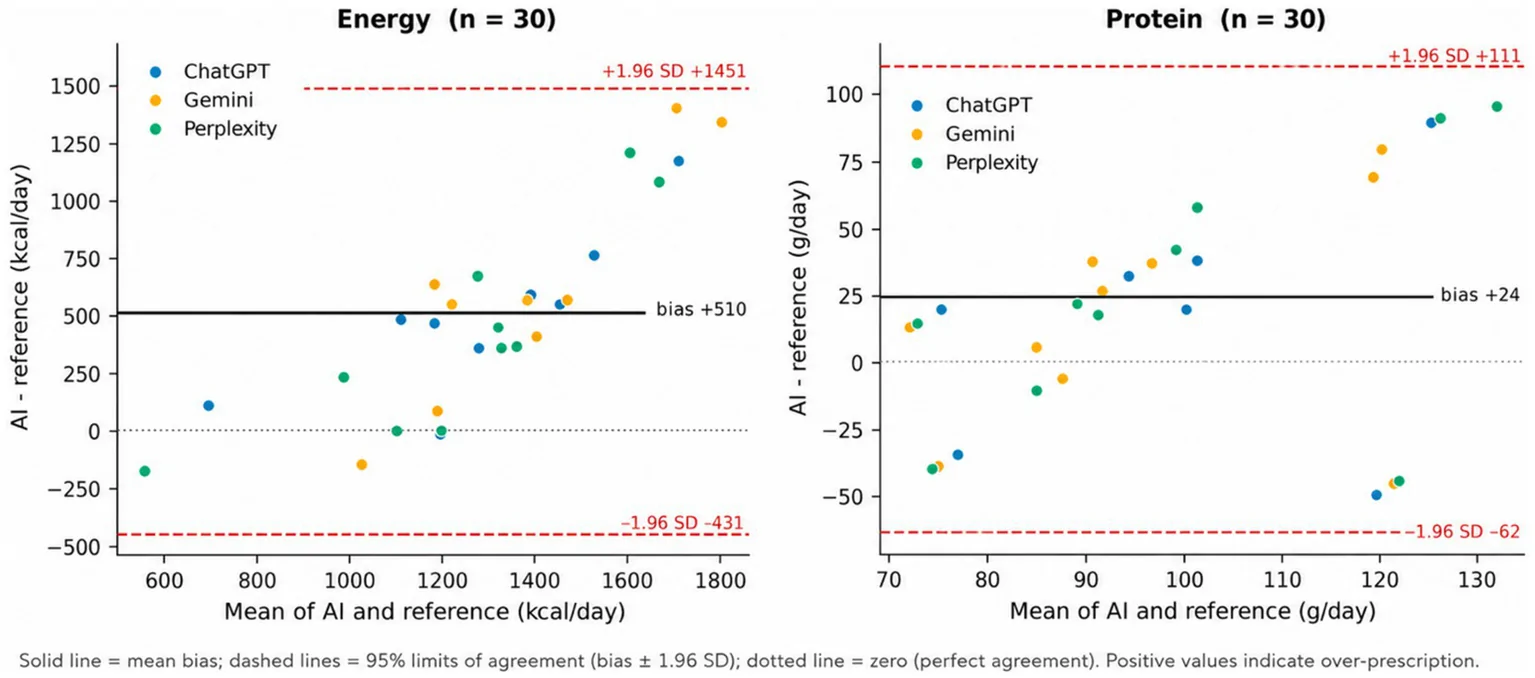
Bland–Altman plots for energy and protein.

Concordance analyses were constrained by incomplete numerical reporting for several electrolytes and micronutrients (Figure 5). Sodium recommendations were explicitly documented in eight out of 10 ChatGPT responses, two out of 10 Gemini responses, and none of the Perplexity responses. Similarly, Perplexity failed to report total fluid volume or sodium values across all evaluated cases, thereby diminishing the number of observations available for quantitative comparison.

**Figure 5:**
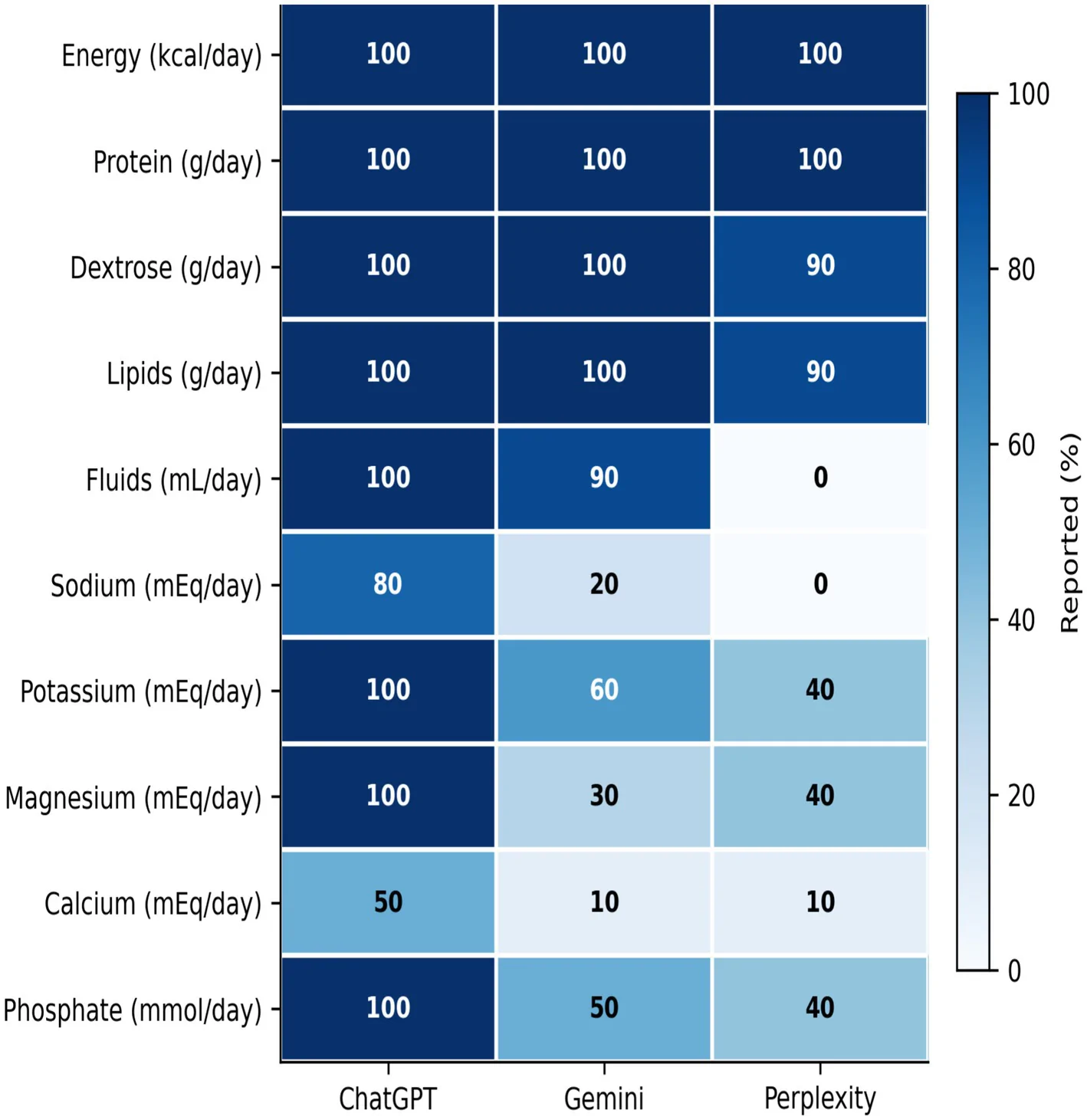
Numerical reporting rate of total parenteral nutrition parameters by chatbot.

## 4 Discussion

This simulation-based benchmarking study comparing AI chatbots to pharmacist-derived reference plans found that the chatbots frequently generated TPN regimens for critically ill adults that were incomplete and, more concerning, unsafe. ChatGPT showed the highest median accuracy and was more complete, but the overall accuracy differences among chatbots were not statistically significant. Although ChatGPT had the highest observed median accuracy, overall accuracy differences among chatbots were not statistically significant (*p* = 0.124). This finding should not be interpreted as evidence of equivalent performance among the evaluated chatbots. Importantly, none of the chatbots evaluated performed well enough to support autonomous TPN prescribing. Each plan contained at least one significant safety issue, nearly 25% presented a critical concern, and only about 10% required no modifications to be deemed acceptable. These findings temper the enthusiasm generated by chatbots’ success on knowledge tests and patient education tasks, revealing that creating a fully complete, internally consistent, safety-critical prescription is a significantly more challenging problem (12–15).

A key finding was the clear difference in performance between macronutrient and micronutrient-related parameters. Chatbots generally performed better in energy, protein, dextrose, and lipid recommendations, whereas substantially poorer performance was observed for electrolytes, micronutrients, and monitoring. These areas require patient-specific adjustments and are less suited to pattern recognition. This is reflected in the error taxonomy: electrolyte, micronutrient, and monitoring mistakes were prevalent across most plans, with trace elements and multivitamins almost never dosed correctly. The gap in completeness was significant (ChatGPT vs. Perplexity rank-biserial r = 1.00), supporting previous findings that chatbots produce fluent but unreliable numerical outputs and that accuracy declines when tasks involve precise, multi-factor reasoning (10, 16). Additionally, Solomon and Laye evaluated several AI chatbots in sports nutrition and reported that their accuracy varied widely, with overall performance being moderate and inconsistent (17).

One significant finding in our study was the consistent presence of major or critical safety issues across all the plans evaluated. Every AI-generated prescription needed pharmacist review before use, and nearly 25% had errors that could cause life-threatening harm if left uncorrected. These findings support earlier concerns about generative AI in healthcare, which can produce convincing but sometimes clinically inaccurate recommendations. Similar safety concerns have been raised in research on AI-generated diets and medication recommendations. Niszczota and colleagues observed that ChatGPT-produced diet plans for individuals with food allergies were mostly accurate but sometimes posed risks, often due to errors in calorie counts and portion sizes (18).

Unlike many decision-support tools, TPN prescribing impacts electrolyte levels, fluid balance, metabolic stability, and nutrition. Therefore, even small numerical errors can lead to serious adverse effects, especially in patients with renal failure, refeeding syndrome, or severe electrolyte imbalances. A study conducted to evaluate the safety of AI responses in suicide-related interactions found that AI responses can sometimes offer false assurances and pose important ethical and safety issues (19).

In medication review and reconciliation, Sridharan and Sivaramakrishnan found that large language models showed useful abilities but also overlooked important drug interactions and made mistakes that could impact patient safety (20).

Additionally, the observed patterns of clinical errors highlight the current limitations of LLMs in the domain of nutritional support. Predominant deficiencies were identified in areas such as electrolyte management, micronutrient supplementation, and monitoring recommendations. Recurrent omissions across all chatbots included trace elements, multivitamin administration, monitoring protocols, and refeeding precautions. While these omissions may appear less conspicuous than inaccuracies in macronutrient calculations, they are nevertheless critical components of safe TPN prescription. Failure to recommend proper monitoring may increase the risk of electrolyte imbalance, micronutrient deficiencies, refeeding syndrome, and delayed detection of metabolic disturbances (6, 21). In addition, inappropriate potassium dosing for patients with renal impairment or on renal replacement therapy could potentially result in life-threatening dyskalemia. Collectively, these findings suggest that current LLMs predominantly generate general nutritional advice, while inadequately addressing safety-critical aspects of individualized TPN management. A study done by Yüceer-Çetiner et al. evaluated AI outputs in answering patient-oriented frequently asked questions about orthognathic surgery and found that AI chatbots vary significantly in their ability to support surgical patient education, carrying a significant risk when delivering that information to patients directly without medical review and supervision (22).

Recent evidence further highlights the variability in the quality and reliability of AI-generated health information across different chatbot platforms and clinical areas. Urtekin and Kartal examined five popular AI chatbots’ responses to common apheresis-related questions, finding notable differences in information quality, reliability, and readability, evaluated using the Ensuring Quality Information for Patients (EQIP) and DISCERN tools as well as readability measures (23). Similarly, Urtekin assessed ChatGPT, Gemini, and Microsoft Copilot for hypertension-related content, revealing performance variations when 11 clinical experts independently reviewed responses with the same assessment tools (24). While these studies mainly focus on patient-oriented health information rather than personalized therapeutic decisions, their results highlight that chatbot performance depends on the platform and task, cautioning against broad generalizations across clinical contexts. This aligns with broader research: a systematic review of 137 studies on LLM-generated health advice shows significant variability in evaluation and reporting, with less than a third addressing ethical, regulatory, and safety concerns (25). A larger review of 761 clinical LLM evaluations stresses the necessity of rigorous reliability and safety assessments before clinical use (26). These issues are especially important for TPN, where recommendations depend on multiple patient-specific clinical and biochemical factors, and where seemingly plausible numerical outputs could have critical safety implications. Collectively, these findings reinforce the importance of task-specific, clinically grounded evaluation and expert assessment of AI systems, particularly when applied to complex, safety-critical clinical decision-making such as individualized TPN prescribing.

This study has notable strengths. To our knowledge, it is among the first to systematically compare several publicly available AI chatbots for pharmacist-led TPN planning in critically ill adults, using standardized clinical cases and pharmacist-created reference plans. Unlike earlier studies that mainly focused on factual accuracy or guideline interpretation, our study included multiple complementary outcome measures, such as rubric-based accuracy, completeness, clinical safety, acceptability, structured error taxonomy, and quantitative concordance. This multidimensional approach offers a more thorough evaluation of AI performance in a complex prescribing task.

Several limitations should be acknowledged. First, the study evaluated only 10 simulated clinical cases, which may not fully reflect the diversity of patients seen in routine critical care settings. This may have reduced statistical power to detect clinically meaningful differences, increased the possibility of type II error, and restricted the generalizability of the findings. Future studies should evaluate larger and more diverse case sets to confirm and extend these results. Second, only publicly available versions of three AI chatbots were tested at a single point in time, and their performance could change as they are updated. Therefore, the study did not measure test–retest reproducibility or version-to-version stability, which the study considers as benchmarking. However, we reported the access date for all AI chatbots. The AI chatbots for healthcare were intended to be used in our study, but they were available only by invitation or were not yet available in our region. Third, the reference TPN plans and subsequent evaluation of chatbot-generated plans were performed by the study investigator. Although we used a predefined guideline-based evaluation instrument to improve consistency, some aspects of TPN prescribing and safety assessment may involve clinical judgment, and multiple clinically acceptable regimens may be possible for an individual case. In addition, using a single evaluator precluded assessment of inter-rater agreement. Future studies should therefore include multiple independent nutrition-support experts and consensus adjudication where appropriate. Fourth, only one standardized prompt was applied per case; different prompting methods could affect model performance. Additionally, we used the “new chat” function and did not allow any conversational thread or case-specific context to carry over between prompts. We also accessed the AI platforms in a way that minimized prior user interaction and potential personalization when possible. Nevertheless, this may not completely eliminate account-level memory, personalization, or other persistent contextual information; therefore, a fully “tabula rasa” model state cannot be guaranteed. Lastly, this was a simulation study that did not assess clinical outcomes, workflow integration, user acceptance, or patient safety in real-world settings.

Future research should focus on examining newer generations of healthcare-oriented LLMs, utilizing larger sets of standardized and real-world clinical cases with independent evaluations by multiple nutrition support experts. Further studies should explore whether methods like retrieval-augmented generation, integrating current ASPEN and ESPEN guidelines, structured prompting, or institution-specific protocols can enhance the safety and consistency of AI-generated TPN recommendations. Additionally, prospective research on human–AI collaborative workflows could clarify the optimal role of LLMs in nutrition support teams, always prioritizing patient safety.

## 5 Conclusion

Among the assessed chatbots, ChatGPT generated the most comprehensive and consistently highest-rated TPN plans. Nonetheless, none of the systems exhibited adequate accuracy, safety, or quantitative concordance for autonomous application. Critical or major issues were identified in every plan, with frequent errors related to electrolytes, micronutrients, monitoring, and refeeding precautions. Currently, AI chatbots may serve as supervised educational or supportive tools for generating TPN plans, but all AI-derived TPN recommendations must undergo thorough review by a well-trained clinical pharmacist or nutrition support specialist prior to clinical implementation at the patient level.

## Data Availability

The original contributions presented in the study are included in the article/Supplementary material, further inquiries can be directed to the corresponding author.
