# Supplementary material for: Clinical evaluation of AI Chatbots’ performance and safety for total parenteral nutrition planning in critically ill patients: a simulation-based benchmarking study
Source: Front Nutr. 2026 Oct 6;13:1969789. doi: 10.3389/fnut.2026.1969789 (PMC13680149; doi:10.3389/fnut.2026.1969789)
Supplement: Supplementary file 1 [file Table_1.DOCX]

## Table S1. Pharmacist-derived reference TPN plans

| Case number | Fluids | Calories Target | Dextrose | Protein | Lipids | Na | K | Mg | Ca | PO4 | Trace elements | Multivitamins |
| --- | --- | --- | --- | --- | --- | --- | --- | --- | --- | --- | --- | --- |
| 1.0 | 1200.0 | 2000.0 | 247.0 | 90.0 | 0.0 | 65.0 | 25.0 | 10.0 | 8.0 | 25.0 | 5.0 | 10.0 |
| 2.0 | 1200.0 | 1875.0 | 194.0 | 72.0 | 20.0 | 70.0 | 35.0 | 12.0 | 8.0 | 40.0 | 5.0 | 10.0 |
| 3.0 | 1440.0 | 1175.0 | 50.0 | 94.0 | 10.0 | 70.0 | 40.0 | 12.0 | 8.0 | 30.0 | 5.0 | 10.0 |
| 4.0 | 1920.0 | 1950.0 | 195.0 | 78.0 | 20.0 | 70.0 | 25.0 | 8.0 | 8.0 | 15.0 | 5.0 | 10.0 |
| 5.0 | 1920.0 | 1800.0 | 95.0 | 144.0 | 20.0 | 70.0 | 30.0 | 8.0 | 8.0 | 15.0 | 5.0 | 10.0 |
| 6.0 | 1920.0 | 2000.0 | 170.0 | 80.0 | 10.0 | 70.0 | 40.0 | 15.0 | 10.0 | 35.0 | 5.0 | 10.0 |
| 7.0 | 2100.0 | 1750.0 | 122.0 | 65.0 | 20.0 | 68.0 | 30.0 | 8.0 | 8.0 | 15.0 | 5.0 | 10.0 |
| 8.0 | 1560.0 | 2200.0 | 167.0 | 82.0 | 20.0 | 75.0 | 20.0 | 10.0 | 8.0 | 25.0 | 5.0 | 10.0 |
| 9.0 | 1200.0 | 2250.0 | 173.0 | 84.0 | 20.0 | 70.0 | 10.0 | 6.0 | 6.0 | 0.0 | 2.5 | 10.0 |
| 10.0 | 1080.0 | 2100.0 | 158.0 | 78.0 | 10.0 | 80.0 | 0.0 | 6.0 | 6.0 | 6.0 | 5.0 | 10.0 |
