# Supplementary material for: Clinical evaluation of AI Chatbots’ performance and safety for total parenteral nutrition planning in critically ill patients: a simulation-based benchmarking study
Source: Front Nutr. 2026 Oct 6;13:1969789. doi: 10.3389/fnut.2026.1969789 (PMC13680149; doi:10.3389/fnut.2026.1969789)
Supplement: Supplementary file 2 [file Table_2.DOCX]

## **Table S2.** ChatGPT-generated TPN plans

| Case number | Did the chatbot generate a complete patient-specific TPN plan? | Total calories | Protein (g/day) | Dextrose (g/day) | Lipids (g/day) | Total fluids (mL/day) | Sodium | Potassium | Magnesium | Calcium | Phosphate | Trace elements | Multivitamins | Monitoring recommendations |
| --- | --- | --- | --- | --- | --- | --- | --- | --- | --- | --- | --- | --- | --- | --- |
| 1 | Yes | 1188 | 110 | 220 | 0 | 1800 | 80-100 | 60-80 | 12-16 | 8-9 | 20-30 | Not mentioned | Not mentioned | Not mentioned |
| 2 | Yes | 1900 | 130 | 220 | 70 | 2000 | 100-140 | 80-120 | 16-24 | 9-15 | 30-40 | Not mentioned | Mentioned but without dosing | Not mentioned |
| 3 | Yes | 750 | 60 | 90 | 20 | 1500 | Missing | 80-120 | 24-32 | Missing | 40-60 | Not mentioned | Not mentioned | Not mentioned |
| 4 | Yes | 1720 | 105 | 230 | 60 | 1800 | 80-120 | 60-80 | 12-16 | 8-12 | 20-30 | Mentioned but without dosing | Mentioned but without dosing | Not mentioned |
| 5 | Yes | 1600 - 1750 | 95 | 220 | 40-50 | 1800 | 80-100 | 50-70 | 10-14 | 8-10 | 20-30 | Not mentioned | Not mentioned | Not mentioned |
| 6 | Yes | 2400-2800 | 150-190 | 250 | 60 | 2000 | 100-140 | 80-120 | 16-24 | Missing | 30-40 | Mentioned but without dosing | Not mentioned | mentioned |
| 7 | Yes | 1345 | 85 | 190 | 40 | 1700 | 80-100 | 40-60 | 10-16 | Missing | 20-30 | Not mentioned | Not mentioned | mentioned |
| 8 | Yes | 1450 | 120 | 180 | 40 | 1800 | Missing | 80-120 | 16-24 | 10-15 | 30-40 | Not mentioned | Not mentioned | Not mentioned |
| 9 | Yes | 2290 | 180 | 250 | 80 | 2200 | 100-140 | 60-100 | 16-24 | Missing | 20-30 | Not mentioned | Not mentioned | Not mentioned |
| 10 | Yes | 1410 | 110 | 180 | 40 | 1500 | 80-120 | 80-120 | 16-24 | Missing | 20-30 | Not mentioned | Not mentioned | mentioned |
