# Supplementary material for: Clinical evaluation of AI Chatbots’ performance and safety for total parenteral nutrition planning in critically ill patients: a simulation-based benchmarking study
Source: Front Nutr. 2026 Oct 6;13:1969789. doi: 10.3389/fnut.2026.1969789 (PMC13680149; doi:10.3389/fnut.2026.1969789)
Supplement: Supplementary file 3 [file Table_3.DOCX]

## **Table S3.** Gemini-generated TPN plans

| Case number | Did the chatbot generate a complete patient-specific TPN plan? | Total calories | Protein (g/day) | Dextrose (g/day) | Lipids (g/day) | Total fluids (mL/day) | Sodium | Potassium | Magnesium | Calcium | Phosphate | Trace elements | Multivitamins | Monitoring recommendations |
| --- | --- | --- | --- | --- | --- | --- | --- | --- | --- | --- | --- | --- | --- | --- |
| 1 | Yes | 1600 | 85 | 110 | 0 | 1000-1200 | Missing | 20-30 | Missing | Missing | 20 | Not mentioned | Not mentioned | Not mentioned |
| 2 | Yes | 1230 | 109 | 150 | 35 | 1800-2000 | Missing | 60-80 | Missing | Missing | 30-40 | Not mentioned | Not mentioned | Not mentioned |
| 3 | Yes | 470 | 56 | 50 | 10 | 1000-1200 | Missing | 60 | 16 | Missing | 30 | Not mentioned | Not mentioned | Not mentioned |
| 4 | Yes | 1745 | 105 | 220 | 45 | 2000-2500 | 80 | 60 | 8 | Missing | 20 | Not mentioned | Not mentioned | Not mentioned |
| 5 | Yes | 1655 | 99 | 210 | 40 | 2000 | Missing | Missing | Missing | Missing | Missing | Mentioned but without dosing | Not mentioned | Not mentioned |
| 6 | Yes | 2400 | 160 | 310 | 50 | 2400 | 100 | 60 | 3 | 1 | 35 | Mentioned | Mentioned | mentioned |
| 7 | Partial | 1490 | 79 | 180 | 35 | 1500-1800 | Missing | 40 | Missing | Missing | Missing | Mentioned but without dosing | Not mentioned | Not mentioned |
| 8 | Partial | 800-1100 | 88 | 100 | 10-15 | 800-1000 | Missing | Missing | Missing | Missing | Missing | Not mentioned | Not mentioned | Not mentioned |
| 9 | Partial | 2466 | 154 | 320 | 60 | 2000-2500 | Missing | Missing | Missing | Missing | Missing | Not mentioned | Not mentioned | Not mentioned |
| 10 | Partial | 1488 | 115 | 180 | 30 | Missing | Missing | Missing | Missing | Missing | Missing | Not mentioned | Not mentioned | Not mentioned |
