# Supplementary material for: Clinical evaluation of AI Chatbots’ performance and safety for total parenteral nutrition planning in critically ill patients: a simulation-based benchmarking study
Source: Front Nutr. 2026 Oct 6;13:1969789. doi: 10.3389/fnut.2026.1969789 (PMC13680149; doi:10.3389/fnut.2026.1969789)
Supplement: Supplementary file 4 [file Table_4.DOCX]

## **Table S4.** Perplexity-generated TPN plans

| Case number | Did the chatbot generate a complete patient-specific TPN plan? | Total calories | Protein (g/day) | Dextrose (g/day) | Lipids (g/day) | Total fluids (mL/day) | Sodium | Potassium | Magnesium | Calcium | Phosphate | Trace elements | Multivitamins | Monitoring recommendations |
| --- | --- | --- | --- | --- | --- | --- | --- | --- | --- | --- | --- | --- | --- | --- |
| 1 | Yes | 1200 | 80 | 220 | 0 | Missing | Missing | 40-60 | 8-12 | 5-9 | 20-30 | Mentioned but without dosing | Mentioned but without dosing | Not mentioned |
| 2 | Yes | 1500 | 130 | 150 | 44 | Missing | Missing | 60-80 | 12-16 | Missing | 30-40 | Not mentioned | Not mentioned | Not mentioned |
| 3 | Yes | 450-500 | 55 | 75 | 10 | Missing | Missing | 60-80 | 12-16 | Missing | 30-50 | Not mentioned | Not mentioned | Not mentioned |
| 4 | Yes | 1370 - 1700 | 100 | 200 | 44 | Missing | Missing | Missing | Missing | Missing | Missing | Mentioned but without dosing | Mentioned but without dosing | Not mentioned |
| 5 | Partial | 1370-1700 | 100 | 200 | 44 | Missing | Missing | 40-60 | 8-12 | Missing | 20-30 | Mentioned but without dosing | Mentioned but without dosing | mentioned |
| 6 | Partial | 2000-2400 | 144-200 | Missing | Missing | Missing | Missing | Missing | Missing | Missing | Missing | Mentioned but without dosing | Not mentioned | Not mentioned |
| 7 | Partial | 1100 | 80 | 150 | 20 | Missing | Missing | Missing | Missing | Missing | Missing | Mentioned but without dosing | Mentioned but without dosing | Not mentioned |
| 8 | Partial | 1100 | 100 | 175 | 0 | Missing | Missing | Missing | Missing | Missing | Missing | Mentioned but without dosing | Mentioned but without dosing | Not mentioned |
| 9 | Partial | 2200 | 180 | 250 | 53 | Missing | Missing | Missing | Missing | Missing | Missing | Not mentioned | Not mentioned | Not mentioned |
| 10 | Partial | 1600 | 120 | 180 | 44 | Missing | Missing | Missing | Missing | Missing | Missing | Not mentioned | Not mentioned | Not mentioned |
