# Supplementary material for: Clinical evaluation of AI Chatbots’ performance and safety for total parenteral nutrition planning in critically ill patients: a simulation-based benchmarking study
Source: Front Nutr. 2026 Oct 6;13:1969789. doi: 10.3389/fnut.2026.1969789 (PMC13680149; doi:10.3389/fnut.2026.1969789)
Supplement: Supplementary file 5 [file Table_5.docx]

## C01 — Pancreatitis + hypertriglyceridemic + AKI

**Demographics:** 48M · 96 kg · 175 cm · BMI 31.3 · IBW 70.7 kg · AdjBW 80.8 kg

**Scenario:** Severe acute pancreatitis secondary to hypertriglyceridemia. NPO with ileus, EN-intolerant (high residuals, distension), day 4. Norepinephrine 0.05 mcg/kg/min. Oliguric AKI.

| **Electrolytes** | Na 138 · K 3.4 · Cl 104 · HCO₃ 18 · Ca 7.6 (corr 8.7) · Mg 1.5 · PO₄ 2.2 · Glucose 220 |
| --- | --- |
| **Renal** | BUN 42 · Cr 1.9 · eGFR 42 · UOP 0.4 mL/kg/hr (oliguric) · RRT none |
| **Liver** | AST 70 · ALT 65 · ALP 130 · GGT 90 · T.bili 1.4 · D.bili 0.6 · Albumin 2.6 · INR 1.3 |
| **Triglycerides** | 1850 |
| **ABG** | pH 7.30 · PaCO₂ 32 · PaO₂ 88 · HCO₃ 16 · BE −8 · Lactate 3.1 · FiO₂ 40% |

## C02 — CRRT (septic shock, CVVHDF)

**Demographics:** 64F · 72 kg · 162 cm · BMI 27.4 · IBW 54.5 kg · AdjBW 61.5 kg

**Scenario:** Urosepsis with septic shock and multiorgan failure. AKI on CVVHDF day 3, deeply catabolic, NPO (ileus). Norepinephrine 0.2 mcg/kg/min + vasopressin.

| **Electrolytes** | Na 134 · K 3.2 · Cl 100 · HCO₃ 24 · Ca 8.0 (corr 9.3) · Mg 1.4 · PO₄ 1.8 · Glucose 165 |
| --- | --- |
| **Renal** | BUN 38 · Cr 2.8 · eGFR <15 · UOP anuric · RRT CVVHDF |
| **Liver** | AST 55 · ALT 40 · ALP 110 · GGT 70 · T.bili 1.1 · D.bili 0.4 · Albumin 2.4 · INR 1.4 |
| **Triglycerides** | 180 |
| **ABG** | pH 7.34 · PaCO₂ 34 · PaO₂ 95 · HCO₃ 21 · BE −4 · Lactate 2.2 · FiO₂ 45% |

## C03 — Refeeding syndrome (low weight, alcohol, ARDS)

**Demographics:** 58M · 47 kg · 172 cm · BMI 15.9 · IBW 68 kg · (below IBW — use actual weight)

**Scenario:** Chronic alcohol use, severe malnutrition, ~3 weeks of negligible intake. Admitted with aspiration pneumonia/moderate ARDS, intubated, NPO, GI intolerance.

| **Electrolytes** | Na 136 · K 2.9 · Cl 98 · HCO₃ 26 · Ca 8.2 (corr 9.6) · Mg 1.3 · PO₄ 1.6 · Glucose 92 |
| --- | --- |
| **Renal** | BUN 10 · Cr 0.6 · eGFR >90 · UOP normal · RRT none |
| **Liver** | AST 95 · ALT 60 · ALP 140 · GGT 220 · T.bili 2.1 · D.bili 1.0 · Albumin 2.3 · INR 1.5 |
| **Triglycerides** | 95 |
| **ABG** | pH 7.46 · PaCO₂ 36 · PaO₂ 78 · HCO₃ 25 · BE +1 · Lactate 1.2 · FiO₂ 50% |

## C04 — Normal post-op (elective esophagectomy)

**Demographics:** 61M · 78 kg · 174 cm · BMI 25.8 · IBW 69.8 kg · AdjBW 73.1 kg

**Scenario:** Elective esophagectomy, anastomosis protected, planned NPO 7 days, otherwise haemodynamically stable.

| **Electrolytes** | Na 139 · K 4.1 · Cl 103 · HCO₃ 25 · Ca 8.9 (corr 9.1) · Mg 2.0 · PO₄ 3.2 · Glucose 132 |
| --- | --- |
| **Renal** | BUN 14 · Cr 0.9 · eGFR 92 · UOP 0.8 mL/kg/hr · RRT none |
| **Liver** | AST 28 · ALT 30 · ALP 88 · GGT 35 · T.bili 0.7 · D.bili 0.2 · Albumin 3.4 · INR 1.1 |
| **Triglycerides** | 118 |
| **ABG** | pH 7.41 · PaCO₂ 40 · PaO₂ 92 · HCO₃ 24 · BE 0 · Lactate 1.1 · FiO₂ 30% |

## C05 — Normal post-op (post-Whipple, prolonged ileus)

**Demographics:** 66M · 72 kg · 170 cm · BMI 24.9 · IBW 66.2 kg · AdjBW 68.5 kg

**Scenario:** Post-pancreaticoduodenectomy day 6, persistent ileus, no enteral access. Mild post-op cholestasis.

| **Electrolytes** | Na 137 · K 3.8 · Cl 102 · HCO₃ 24 · Ca 8.6 (corr 9.3) · Mg 1.9 · PO₄ 2.9 · Glucose 145 |
| --- | --- |
| **Renal** | BUN 16 · Cr 1.0 · eGFR 84 · UOP 0.7 mL/kg/hr · RRT none |
| **Liver** | AST 45 · ALT 50 · ALP 160 · GGT 120 · T.bili 1.6 · D.bili 0.9 · Albumin 3.1 · INR 1.2 |
| **Triglycerides** | 130 |
| **ABG** | pH 7.43 · PaCO₂ 38 · PaO₂ 90 · HCO₃ 25 · BE +1 · Lactate 1.0 · FiO₂ 28% |

## C06 — Burn (40% TBSA, hypermetabolic)

**Demographics:** 35M · 80 kg · 180 cm · BMI 24.7 · IBW 75.2 kg

**Scenario:** 40% TBSA burn, hypermetabolic and protein-losing, EN-supplemented but insufficient, NPO for procedures.

| **Electrolytes** | Na 142 · K 3.8 · Cl 108 · HCO₃ 22 · Ca 7.8 (corr 8.6) · Mg 1.5 · PO₄ 2.2 · Glucose 180 |
| --- | --- |
| **Renal** | BUN 28 · Cr 1.1 · eGFR 80 · UOP 0.8 mL/kg/hr · RRT none |
| **Liver** | AST 55 · ALT 45 · ALP 120 · GGT 70 · T.bili 1.0 · D.bili 0.4 · Albumin 2.2 · INR 1.2 |
| **Triglycerides** | 190 |
| **ABG** | pH 7.36 · PaCO₂ 34 · PaO₂ 94 · HCO₃ 20 · BE −4 · Lactate 2.0 · FiO₂ 40% |

## C07 — Pancreatitis (gallstone, normal TG, no AKI)

**Demographics:** 45F · 70 kg · 160 cm · BMI 27.3 · IBW 52.7 kg · AdjBW 59.6 kg

**Scenario:** Gallstone pancreatitis with obstructive/cholestatic picture. NPO, EN-intolerant, preserved renal function.

| **Electrolytes** | Na 137 · K 3.6 · Cl 101 · HCO₃ 22 · Ca 8.0 (corr 8.6) · Mg 1.7 · PO₄ 2.6 · Glucose 150 |
| --- | --- |
| **Renal** | BUN 18 · Cr 1.0 · eGFR 70 · UOP 0.8 mL/kg/hr · RRT none |
| **Liver** | AST 120 · ALT 140 · ALP 210 · GGT 260 · T.bili 2.8 · D.bili 1.9 · Albumin 3.2 · INR 1.1 |
| **Triglycerides** | 160 |
| **ABG** | pH 7.36 · PaCO₂ 36 · PaO₂ 90 · HCO₃ 21 · BE −3 · Lactate 1.8 · FiO₂ 30% |

## C08 — Pancreatitis (necrotizing, abdominal compartment, pressors)

**Demographics:** 50M · 88 kg · 178 cm · BMI 27.8 · IBW 73.4 kg · AdjBW 79.2 kg

**Scenario:** Necrotizing pancreatitis with intra-abdominal hypertension and distributive shock on norepinephrine. NPO.

| **Electrolytes** | Na 136 · K 4.4 · Cl 105 · HCO₃ 17 · Ca 7.4 (corr 8.6) · Mg 1.5 · PO₄ 2.0 · Glucose 198 |
| --- | --- |
| **Renal** | BUN 40 · Cr 1.7 · eGFR 48 · UOP 0.4 mL/kg/hr · RRT none |
| **Liver** | AST 65 · ALT 55 · ALP 120 · GGT 80 · T.bili 1.5 · D.bili 0.7 · Albumin 2.5 · INR 1.4 |
| **Triglycerides** | 240 |
| **ABG** | pH 7.28 · PaCO₂ 30 · PaO₂ 82 · HCO₃ 15 · BE −10 · Lactate 4.2 · FiO₂ 50% |

## C09 — CRRT (trauma rhabdomyolysis, CVVH, catabolic)

**Demographics:** 34M · 90 kg · 182 cm · BMI 27.2 · IBW 77 kg · AdjBW 82.2 kg

**Scenario:** Polytrauma with rhabdomyolysis (CK markedly elevated), AKI on CVVH, hypercatabolic, NPO.

| **Electrolytes** | Na 139 · K 4.0 · Cl 103 · HCO₃ 22 · Ca 7.8 (corr 8.6) · Mg 1.6 · PO₄ 4.8 · Glucose 150 |
| --- | --- |
| **Renal** | BUN 45 · Cr 3.2 · eGFR <15 · UOP oliguric · RRT CVVH |
| **Liver** | AST 320 · ALT 110 · ALP 90 · GGT 50 · T.bili 0.9 · D.bili 0.3 · Albumin 2.8 · INR 1.3 |
| **Triglycerides** | 165 |
| **ABG** | pH 7.33 · PaCO₂ 34 · PaO₂ 96 · HCO₃ 20 · BE −5 · Lactate 2.5 · FiO₂ 40% |

## C10 — CRRT (cardiogenic shock, fluid overload, CVVHDF)

**Demographics:** 68M · 84 kg · 172 cm · BMI 28.4 · IBW 68 kg · AdjBW 74.4 kg

**Scenario:** Cardiogenic shock with congestive hepatopathy. AKI on CVVHDF for volume control, NPO.

| **Electrolytes** | Na 133 · K 4.2 · Cl 98 · HCO₃ 20 · Ca 8.2 (corr 9.0) · Mg 1.8 · PO₄ 3.0 · Glucose 160 |
| --- | --- |
| **Renal** | BUN 52 · Cr 2.6 · eGFR <20 · UOP anuric · RRT CVVHDF |
| **Liver** | AST 90 · ALT 85 · ALP 130 · GGT 95 · T.bili 2.2 · D.bili 1.0 · Albumin 2.9 · INR 1.6 |
| **Triglycerides** | 175 |
| **ABG** | pH 7.31 · PaCO₂ 32 · PaO₂ 90 · HCO₃ 18 · BE −7 · Lactate 3.5 · FiO₂ 50% |

**Prompt used for all cases and AI chatbots: “Formulate a total parenteral nutrition plan for this patient according to ASPEN/ESPEN guidelines.”**
