# Supplementary material for: Clinical evaluation of AI Chatbots’ performance and safety for total parenteral nutrition planning in critically ill patients: a simulation-based benchmarking study
Source: Front Nutr. 2026 Oct 6;13:1969789. doi: 10.3389/fnut.2026.1969789 (PMC13680149; doi:10.3389/fnut.2026.1969789)
Supplement: Supplementary file 6 [file Table_6.docx]

**Table 1. Accuracy assessment**

| **Domain** | **Criterion** | **Score = 1** | **Score = 0** |
| --- | --- | --- | --- |
| Energy | Caloric target appropriateness | Recommended caloric target judged appropriate for the case according to the predefined pharmacist reference and applicable ASPEN/ESPEN recommendations | Target judged inappropriate |
| Protein | Protein target accuracy | Protein recommendation judged appropriate for the case | Recommendation judged inappropriate |
| Fluid | Fluid volume appropriateness | Proposed TPN fluid volume appropriate for the patient's clinical/fluid status | Volume inappropriate |
| Dextrose | Dextrose rate accuracy | Dextrose amount/rate appropriate for the case | Dextrose amount/rate inappropriate |
| Lipids | Lipid dosing accuracy | Lipid dose appropriate for the case | Lipid dose inappropriate |
| Electrolytes | Potassium appropriateness | Potassium recommendation appropriate for the patient's clinical status | Inappropriate potassium recommendation |
| Electrolytes | Sodium appropriateness | Sodium recommendation appropriate for the patient's clinical status | Inappropriate sodium recommendation |
| Electrolytes | Magnesium appropriateness | Magnesium recommendation appropriate for the patient's clinical status | Inappropriate magnesium recommendation |
| Electrolytes | Calcium appropriateness | Calcium recommendation appropriate for the patient's clinical status | Inappropriate calcium recommendation |
| Electrolytes | Phosphate appropriateness | Phosphate recommendation appropriate for the patient's clinical status | Inappropriate phosphate recommendation |
| Micronutrients | Micronutrient selection accuracy | Micronutrient recommendation appropriate | Inappropriate micronutrient recommendation |
| Acid–base | Acid–base considerations | Relevant acid–base issues appropriately considered | Relevant acid–base issues not appropriately considered |

**Table 2. Completeness assessment**

| **Criterion** | **Score = 1** | **Score = 0** | **N/A** |
| --- | --- | --- | --- |
| Total daily calories stated | Explicitly reported | Not reported | — |
| Protein dose included | Explicitly reported | Not reported | — |
| Dextrose dose included | Explicitly reported | Not reported | — |
| Lipid dose included | Explicitly reported | Not reported | — |
| TPN volume specified | Explicitly reported | Not reported | — |
| Sodium included | Explicitly reported | Not reported | — |
| Potassium included | Explicitly reported | Not reported | — |
| Magnesium included | Explicitly reported | Not reported | — |
| Phosphate included | Explicitly reported | Not reported | — |
| Calcium included | Explicitly reported | Not reported | — |
| Trace elements included | Explicitly addressed | Not addressed | — |
| Multivitamins included | Explicitly addressed | Not addressed | — |
| Refeeding precautions addressed | Appropriate precautions addressed | Not addressed when required | Case not requiring refeeding precautions |
| Insulin recommendation addressed | Addressed when clinically indicated | Not addressed when required | Not clinically applicable |
| Fluid restriction addressed | Addressed when clinically indicated | Not addressed when required | Not clinically applicable |
| Infusion rate/total hours specified | Explicitly reported | Not reported | — |
| Monitoring plan listed | Monitoring recommendations provided | Not provided | — |

**Table 3. Safety assessment**

| **Assessment** | **Categories** |
| --- | --- |
| Clinically significant safety concern present? | Yes / No |
| Error severity | No clinically significant error / Minor / Moderate / Major / Critical |
| Error domain | Calories / Protein / Dextrose / Lipids / Electrolytes / Fluid / Micronutrients / Monitoring / Refeeding / Other |
| Error description | Free-text description of the identified clinical error |

**Table 4. Clinical acceptability**

| **Classification** | **Operational interpretation** |
| --- | --- |
| Acceptable without modification | Plan could be used without clinically relevant pharmacist modification |
| Acceptable with minor modifications | Overall plan acceptable but requires limited correction/modification |
| Rejected / Not acceptable | Clinically important deficiencies require substantial modification before use |
