# Supplementary material for: Clinical evaluation of AI Chatbots’ performance and safety for total parenteral nutrition planning in critically ill patients: a simulation-based benchmarking study
Source: Front Nutr. 2026 Oct 6;13:1969789. doi: 10.3389/fnut.2026.1969789 (PMC13680149; doi:10.3389/fnut.2026.1969789)
Supplement: Supplementary file 7 [file Table_7.docx]

**Table S7. ASPEN-based criteria and acceptable ranges used for accuracy assessment**

| **Accuracy criterion** | **ASPEN-based acceptable range** |
| --- | --- |
| **Energy** | 20–30 kcal/kg/day for critically ill adults, depends on the patient condition |
| **Protein** | 1.2–2.5 g/kg/day for critically ill adults, depends on the patient condition |
| **Dextrose/GIR** | <4 mg/kg/min in critically ill patients |
| **Lipids** | <1 g/kg/day in critically ill patients |
| **Fluid volume** | Individualized; in critically ill patients, use the minimum volume necessary to provide required macronutrients, with adjustment for fluid status and restriction |
| **Sodium** | 1–2 mEq/kg/day |
| **Potassium** | 1–2 mEq/kg/day |
| **Magnesium** | 8–20 mEq/day |
| **Calcium** | 10–15 mEq/day |
| **Phosphate** | 20–40 mmol/day |
| **Trace elements** | Zinc 3–5 mg/day; copper 0.3–0.5 mg/day; manganese 55 mcg/day; selenium 60–100 mcg/day; chromium ≤10 mcg/day |
| **Multivitamins** | Full daily adult parenteral multivitamin dose unless adequately supplied enterally/orally or otherwise clinically modified |
| **Acid–base balance** | Chloride and acetate adjusted as needed to maintain acid–base balance |

Score interpretation: 1 = within the recommended range or appropriately individualized for the case; 0 = outside the range without clinical justification
